# OP Pesticides, Organic Diets, and Children’s Health

**DOI:** 10.1289/ehp.114-a572a

**Published:** 2006-10

**Authors:** Robert I. Krieger, James J. Keenan, Yanhong Li, Helen M. Vega

**Affiliations:** Personal Chemical Exposure Program, Department of Entomology, University of California, Riverside, Riverside, California, E-mail: bob.krieger@ucr.edu

The importance of “judicious use of language in regard to public communication of pesticide health risks” (Lu et al. 2006b) is clearly recognized and acknowledged in recent letters from Avery (2006) and Lu et al. (2006b). Their correspondence concerned perceptions of risk conveyed by the article “Organic Diets Significantly Lower Children’s Dietary Exposure to Organophosphorous Pesticides,” published by Lu et al. (2006a). My concern is more fundamental than the need for effective communication and the stated “public misunderstanding of this important issue” (Lu et al. 2006b). I believe the primary issue concerns science and how we accumulate knowledge.

There is no guarantee that judicious use of language can prevent misunderstanding of even the most rigorous and carefully performed studies. It is important, however, to put the results into the existing scientific and regulatory contexts. Lu et al. (2006a) noted that “the paucity of exposure data renders the debate over pesticide-related health risks in children controversial.” Curl et al. (2003) stated that “reduction of children’s risk from pesticides requires an understanding of the pathways by which exposure occurs.” The primary objective of the longitudinal study by Lu et al. (2006a) was determination of “overall pesticide exposure in a group of elementary school-age children.” The authors reported that children who consumed organic diets eliminated (via urine) nondetectable amounts of organophosphorous (OP) insecticide metabolites. The finding supports the consensus that the diet is the predominant source of OP compounds and OP metabolites excreted in urine (Barr et al. 2004; Duggan et al. 2003; Krieger et al. 2003).

Lu et al. (2006a) claimed “a convincing demonstration of the ability of organic diets to reduce children’s OP pesticide exposure and the health risks that may be associated with these exposures.” When the study was developed and throughout the period of data collection, analysis, and publication by the University of Washington investigators, there could be no doubt that dietary exposures were very low or miniscule relative to acute toxicity (Curl et al. 2003). Indeed, it is intuitive that the change in diet reduced OP metabolite elimination in urine. If this were not the case, one might expect parked cars to get speeding tickets.

Specific health risks have never been associated with such miniscule insecticide exposures. If risk is defined as the likelihood of an adverse effect in an exposed population, the risk of neurotoxicity caused by these dietary OP exposure(s) is zero; that is, disease has not been observed in the population who consumes food that sometimes contains OP pesticides or OP metabolite residues (Krieger et al. 2003). Back-calculated OP exposures are well below the experimental lowest observed adverse effect level (LOAEL), the estimated no observed adverse effect level (NOAEL), and the regulatory reference dose (RfD) for neurotoxicity of any OP insecticide used in crop protection (Barr et al. 2004; Duggan et al. 2003; Fenske et al. 2000). The research is misrepresented with respect to its relevance to risk reduction (that is the point of the fundamental “observed” in the LOAEL and the NOAEL upon which RfDs are based).

With zero cases of disease in the population exposed to dietary OP pesticide, the numerator of measurements of risk such as odds ratios or relative risk is also zero. As a result, measured risk of acute neurotoxicity is zero. The axiomatic truth that “dose determines a poison” and its corollary that “there is a safe level of everything” must both be considered in responsible risk communication. Careful choice of words may sometimes prevent misunderstanding of health research reports, but more importantly our common understanding and well-being require that we clearly distinguish chemical exposure and health risk. Lu et al. (2006a) wrote,

We were able to demonstrate that an organic diet provides a dramatic and immediate protective effect against exposure to organophosphorus pesticides that are commonly used in agricultural production.

Their findings are expected rather than dramatic, and the term “protective” in reference to a no observed effect exposure is misleading at best. Effective communication requires awareness that potential impacts of conjecture about matters of health and pesticides likely include heightened anxiety and fear, and may prompt misallocation of resources as some persons pursue something less than zero risk—a point where scientific evidence and mystical, supernatural beliefs must be distinguished.
